# Racial/ethnic/gender-based Disparities in Health Trajectories among American Older Adults

**DOI:** 10.1093/geroni/igaa057.3295

**Published:** 2020-12-16

**Authors:** Dexia Kong, Peiyi Lu, Joan Davitt, Mack Shelley

**Affiliations:** 1 Rutgers University, New Brunswick, New Jersey, United States; 2 Iowa State University, Ames, Iowa, United States; 3 University of Maryland, Baltimore, Baltimore, Maryland, United States

## Abstract

Numerous studies have examined racial/ethnic- or gender-based disparities in health. However, few examined health outcomes based on a combination of individuals’ race, ethnicity, and gender. Guided by an intersectionality framework, this study explores racial/ethnic/gender-based differences in older adults’ health trajectories over a ten-year period. Longitudinal data from the Health and Retirement Study (2004-2014) were used (n=16,654). Older adults (65+) were stratified into six groups based on their race, ethnicity, and gender, including (1) Non-Hispanic (NH) White Men; (2) NH White Women; (3) NH Black Men; (4) NH Black Women; (5) Hispanic Men; and (6) Hispanic Women. Growth curve models were used to examine the trajectories of three health indicators over time, including cognitive function, physical function (i.e. the sum of activities of daily living and instrumental activities of daily living), and depressive symptoms. The results indicated that NH White men and women outperformed racial/ethnic minority groups in cognition and physical function trajectories. Females in all racial/ethnic groups had more depressive symptoms but better cognition than their male counterparts. Hispanic women reported the most depressive symptoms. Hispanic women and NH Black women had the poorest physical function. NH Black men/women had the lowest cognition. Study findings highlighted the utility of an intersectionality framework in understanding health disparities in later life. Multiple social identities intersect with each other and generate protective and/or risk effects on cognitive, mental, and physical health status. Multilevel intervention strategies are warranted to close the health equity gap among various marginalized population groups.

